# Orally delivered chitosan-based *Salmonella* subunit nanovaccine provides protective immunity against colonization by *Salmonella* Enteritidis in adult layer chickens

**DOI:** 10.3389/fimmu.2026.1879692

**Published:** 2026-08-05

**Authors:** Raksha Suresh, Shekoni Olaitan Comfort, Sara Dolatyabi, Jennifer Schrock, Mithilesh Singh, Stephen A. Akinola, Gourapura J. Renukaradhya

**Affiliations:** Center for Food Animal Health, Department of Animal Sciences, College of Food, Agricultural, and Environmental Sciences, The Ohio State University, Wooster, OH, United States

**Keywords:** chitosan, layer chickens, nanoparticle vaccine, oral delivery, *Salmonella*

## Abstract

**Introduction:**

*Salmonella* contamination in poultry meat and eggs remains the leading cause of foodborne illnesses in humans. Particularly, *Salmonella* Enteritidis has been frequently identified as a primary source of contamination in eggs. This study was designed to evaluate the effectiveness of a subunit *Salmonella* nanovaccine in adult layers.

**Methods:**

The vaccine was formulated using the mannose-conjugated chitosan nanoparticles (mChitosan-NP) delivery platform, incorporating *S.* Enteritidis derived outer membrane proteins (OMP) and flagella (Fla) entrapped in mChitosan-NP, and surface-coated with Fla [mChitosan (OMP+Fla)/Fla-NP]. This candidate nanovaccine was administered orally to pullets, raised from day 0 of hatching to adult egg laying age. To determine the dose-dependent efficacy of the nanovaccine, three different doses of antigens (200, 50, and 10 microgram) entrapped in NP were administered four times orally at 4, 10, 16, and 18 weeks of age and challenged at age 25 weeks with 10^9^ CFU *S.* Enteritidis. Necropsies were performed at 4 and 8 days post-challenge (DPC) infection, and challenge *Salmonella* load in the cecum, reproductive tract, and eggs was assessed.

**Results and discussion:**

Our data revealed that the highest vaccine dose (200 microgram per bird) resulted in a 2.7 log reduction in cecal *S. Enteritidis* load at DPC4 compared to the control group. Moreover, in the same group, *Salmonella* was undetectable in the upper and lower reproductive tracts on direct culture method. Flow cytometric analysis revealed an increased frequency of activated recall B cell response. Additionally, enhanced antigen-specific antibody responses were detected at both mucosal and systemic sites, particularly at DPC8. Furthermore, enhanced OMP-specific IgY response in the egg yolk was detected in the nanovaccine-administered birds. Overall, the immune response data were positively associated with the vaccine’s efficacy, suggesting that the oral administration of the *Salmonella* nanovaccine has the potential to reduce the risk of *Salmonella* transmission in eggs.

## 1 Introduction

According to the recent 2019 CDC report, 1.3 million illness, 12,500 hospitalizations, and 238 deaths among people had been associated with salmonellosis (1). Poultry is one of the major sources of *Salmonella* infection in humans; thus, an effective vaccination strategy in poultry is warranted. Currently, both genetically modified live *Salmonella* Typhimurium vaccine administered orally and killed injectable vaccine are in use in poultry to mitigate *S*. Enteritidis which causes food poisoning in humans. Poultry eggs are a major source of *Salmonella* for humans. In 2018, 45.6% of the reported salmonellosis outbreaks in the European Union were linked to consumption of contaminated poultry eggs and their products (2).

Globally, egg production has increased dramatically from the 1960s to the 2020s and is expected to rise substantially (3). Although it is estimated that only one in every 20,000 eggs is contaminated with *Salmonella*, the recent outbreaks highlight that this pathogen remains a significant public health concern (4). Decontamination of eggs to eliminate *Salmonella* is fairly a simple process involving pasteurization. However, only 3% of commercial eggs undergo pasteurization because the conventional pasteurization of intact eggs is a lengthy process, typically requiring eggs to be submerged in hot water for more than 57 min (5). Therefore, vaccination of poultry remains a preferred strategy for controlling salmonellosis in humans. In field conditions, killed vaccines like Layermune SE are used widely, but these are not administered until the age of 12 weeks in pullets. This vaccine is administered subcutaneously multiple times, making the process highly labor-intensive and stressful to birds (6). Since *Salmonella* colonization often occurs in early life, there is a risk of birds becoming carriers before vaccination. Although the live vaccines induce a strong cell-mediated immunity essential for clearance of intracellular *Salmonella*, the vaccine strains usually contain undefined mutations in one or more genes, which may revert to a virulent form, posing safety concerns (7).

There are two possible routes by which *Salmonella* can contaminate eggs. One is horizontal transmission, where contamination occurs through the eggshell after contact with contaminated droppings after the egg is laid (8). The other route is vertical transmission, whereby the egg components get contaminated while the egg is formed in the reproductive tract (9, 10). Anatomically, the oviduct of a laying hen is divided into the infundibulum, magnum, isthmus, and shell gland, with each part contributing differently to egg contamination: infection in the infundibulum affects the yolk membranes, whereas infection in the magnum, isthmus, and shell gland affects the albumen, shell membranes, and eggshell, respectively (11).

Although there are over 2,500 serovars of *Salmonella* identified, *S.* Enteritidis remains the most prevalent serovar detected in poultry eggs (12). There are several critical factors that affect the efficacy of *Salmonella* vaccine in layers. One major challenge is the extended lifespan of layers, yet few studies have evaluated vaccine effectiveness from the chick stage through the egg laying period. Additionally, most layer farms house multi-age birds, simultaneously making it difficult to implement an “all in, all out” management system. This limits the ability to thoroughly clean and disinfect the farm between production cycles. Moreover, older birds can transmit *Salmonella* to younger chicks (13). With the introduction of the FDA (US Food and Drug Administration) Egg Rule, it has become increasingly critical for producers to control *Salmonella* contamination in layers. If any poultry environmental testing yields a *Salmonella*-positive result in the farm premises, producers are required to conduct extensive egg testing, which can be costly or divert the eggs to liquid processing for the remainder of the production cycle (14).

Live attenuated *S.* Typhimurium spray/oral vaccine is commonly used in the poultry industry with considerable success in reducing *S.* Enteritidis infections (15). However, live *S.* Typhimurium can also colonize the reproductive tract and contributes to shedding in eggs, but *S.* Enteritidis is more frequently associated with contaminated egg contents than *S*. Typhimurium because *S.* Enteritidis possesses intrinsic traits that enable it to better survive in the antibacterial environment of the oviduct and eggs (11, 16). Furthermore, there are concerns regarding the persistence and shedding of *S.* Typhimurium vaccine strains for long periods, along with potential release to the environment and contamination of the human food chain (17–20). In this regard, our laboratory has developed and evaluated orally delivered nanovaccine platforms using chitosan and polyanhydride particles. Particularly, chitosan is a manufacturer-friendly vaccine delivery system shown to induce protective immunity in orally vaccinated birds (21–23).

In this study, we evaluated the efficacy of our optimized chitosan-based nanoparticle subunit *S.* Enteritidis vaccine (24, 25) for the first time in adult layers. The birds were raised from hatchling to the adult egg laying stage and challenged with *S.* Enteritidis when the egg production reached 50% in layers. A key feature of our subunit nanovaccine is the inclusion of two components—chitosan and mannose. Chitosan is a natural polysaccharide derived from chitin. It exhibits strong electrostatic interactions with negatively charged mucosal surfaces containing molecules such as sialic acid on epithelial cells (26). Thus, due to its mucoadhesive properties, it promotes prolonged mucosal contact time and enhances immune stimulation. Mannose is a monosaccharide that serves as a ligand for the mannose receptor expressed on dendritic cells and macrophages. This targeted oral vaccination strategy facilitates the activation of both antigen-specific B and T cells and promotes the production of specific secretory (s) IgA antibodies in the intestines, thus contributing to robust mucosal immunity (27–29). This study was designed to address the following objectives: (i) optimize the dose of vaccine antigens [outer membrane proteins (OMP) and flagella (Fla) of *S.* Enteritidis] entrapped in mannose chitosan-NP required to substantially decrease the challenge *Salmonella* load both in the intestines and reproductive tract of layers and (ii) evaluate the immune correlates of protection elicited by the *Salmonella* subunit nanovaccine in adult birds.

## 2 Materials and methods

### 2.1 Extraction of outer membrane proteins

The procedures for the extraction of vaccine antigens, namely, outer membrane proteins (OMP) and flagella proteins (Fla), of *S.* Enteritidis were followed as described earlier (30). Briefly*, S.* Enteritidis was cultured in Tryptic soy broth (TSB) until the optical density (OD) reached 1.0. The broth was centrifuged, and the resulting bacterial cell pellet was washed in Tris-HCl buffer. The pellet was frozen overnight at -80°C, thawed, and heated at 75°C for 10 min in a water bath to inactivate the bacteria. The killed bacterial cells were sonicated (VCX-500, Sonics, CT, USA) using a 3-mm microtip at 40% maximum amplitude, with 15-s pulses on and 45-s intervals off for five cycles, and centrifuged; then, the supernatant was collected. The supernatant was ultracentrifuged at 108,726 × *g* for 30 min (Optima L-100 XP Ultracentrifuge, Beckman Coulter, CA, USA), and the resulting pellet was washed with 10 mM Tris-HCl containing 2% Triton X-100 and incubated for 30 min. The pellet was ultracentrifuged again at 100,000 × *g* for 2 to 3 h, and the final pellet was dissolved in 1× PBS. The protein concentration was estimated by using Micro BCA (Bicinchoninic acid) Protein Assay Kit (ThermoFisher Scientific, MA, USA). The protein was lyophilized using FreeZone 4.5 Freeze Dryer (Labconco, MO, USA), with 5% sucrose as a cryoprotectant, and stored at -20 °C.

### 2.2 Extraction of flagella (Fla)

*S*. Enteritidis was cultured in brain heart infusion broth (BHI). Once the OD of the broth reached between 0.6 and 0.8, the bacterial cells were harvested by centrifugation at 4,000 RPM. The resulting pellet was washed with 1× PBS and treated with 3 M potassium thiocyanate in 1× PBS for 2 h at 4 °C under continuous magnetic stirring. Following treatment, the cells were subjected to ultracentrifugation at 35,000 × *g* for 30 min (Optima L-100 XP Ultracentrifuge, Beckman Coulter, CA, USA). The supernatant containing flagellin was dialyzed against 1× PBS and subsequently against Milli-Q water for 12 h each. The dialyzed protein concentration was estimated by using Micro BCAProtein Assay Kit (ThermoFisher Scientific, MA, USA). The protein was lyophilized using FreeZone 4.5 Freeze Dryer (Labconco, MO, USA), with 5% sucrose as a cryoprotectant, and stored at -20 °C (24).

### 2.3 Preparation of mannose-chitosan-based *Salmonella* nanovaccine

The mannose-modified chitosan-NP entrapped with OMP+Fla [mChitosan (OMP+Fla)/Fla-NP or mCS(OMP+Fla)] was prepared using the ionic gelation method as previously described (25, 31, 32). Briefly, lyophilized mannose-conjugated chitosan powder (447 mg) was added into 447 mL of Milli-Q water under magnetic stirring, and the pH was adjusted to 4.3. To this preparation, 44.7 mg of extracted *S.* Enteritidis proteins (22.35 mg each of OMP and Fla) dissolved in 10 mM 3-(-N-morpholino) propanesufonic acid (MOPS) was added dropwise using an insulin syringe under magnetic stirring. Next, 112 mg of sodium tripolyphosphate (TPP) dissolved in Milli Q water was added dropwise to the abovementioned solution under magnetic stirring. To surface-coat the NP vaccine with Fla, 11.2 mg of Fla protein was added dropwise under continuous magnetic stirring. The formulated nanoparticle solution was centrifuged at 10,976 × *g* for 30 min, and then the pellet was collected. Centrifugation was repeated, and the pellets were pooled and resuspended in Milli-Q water and sonicated. The vaccine formulation was lyophilized using FreeZone 4.5 Freeze Dryer (Labconco, MO, USA). The supernatant was used to check the entrapment efficiency of OMP and Fla by using Micro BCA Protein Assay Kit (ThermoFisher Scientific, MA, USA). This procedure was carried out in multiple small batches to facilitate high entrapment efficiency, after which all the batches were pooled (24).

### 2.4 Characterization of mCS(OMP+Fla)/Fla

The particle size and size distribution of mCS(OMP+Fla)/Fla vaccine was characterized using the Zetasizer (Zetasizer Pro, Malvern Panalytical, UK). The vaccine was diluted and loaded onto the Zetasizer and characterized for particle size, zeta potential, and polydispersity index (PDI).

### 2.5 Immunization and challenge studies in chicken

**Figure 1:**
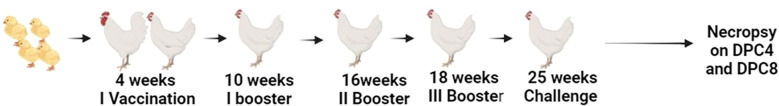
Experimental design of the study.

To begin with, twice the required number of specific-pathogen-free (SPF) White Leghorn layer chicken eggs were incubated because only pullets were needed for this trial. Both cockerels and pullets received the first vaccination at 4 weeks of age. The cockerels were completely removed from the trial by 6–8 weeks of age, and only pullets were retained. Subsequent booster vaccinations were given at 10, 16, and 18 weeks of age. By 25 weeks of age, when the flock had reached at least 50% egg production, the hens were challenged with 1 × 10^9^ colony-forming units (CFU)/bird of *S.* Enteritidis through an oral gavage. Necropsies were performed at 4 and 8 days post-challenge (DPC4 and DPC8). During necropsy, cecum was collected to enumerate the colonization of *S.* Enteritidis. Reproductive tract (lower and upper oviduct) sample was collected for the qualitative testing of *Salmonella*. In addition, bile samples were collected to measure specific immunoglobulin Y (IgY) and secretory immunoglobulin A (sIgA) titers, blood was collected for serum IgY estimation, and cloacal swab and small intestinal wash were collected to evaluate mucosal sIgA. Spleen was collected for quantitative analysis of immune cells. Different treatment groups with their sample size are provided in **Table 1**. The experimental design is provided in **Figure 1**.

**Table 1:** Treatment groups.

Group	Vaccine received	Challenge	Number of birds on DPC4	Number of birds on DPC8
1	Mock	No	6	5
2	mCS- NPs	Yes	6	6
3	OMP and Fla 200µg	Yes	6	6
4	mCS (OMP+Fla)/Fla-200 µg	Yes	6	5
5	mCS (OMP+Fla)/Fla-50 µg	Yes	6	6
6	mCS (OMP+Fla)/Fla-10 µg	Yes	6	6

### 2.6 Challenge bacterial enumeration

#### 2.6.1 Cecal bacterial enumeration

##### 2.6.1.1 Enumeration through standard plate count method

To determine the challenge *S.* Enteritidis (resistant to novobiocin and nalidixic acid) load in the intestines of chickens, cecum from each bird was collected in a separate Whirl-Pak bag during necropsy. The cecum was weighed and diluted 1:5 in PBS (w/v). The cecal contents were manually squeezed, serially diluted 10-fold, plated on Xylose Lysine Tergitol 4 (XLT4) plates supplemented with novobiocin and nalidixic acid (20 microgram/ml), and incubated at 37°C for 24 h. Black colonies were enumerated, and the results were expressed as log CFU/g of cecal content. The negative samples by direct plating were enriched in tetrathionate broth by incubating at 37°C for 24 h and then plated on XLT4 agar plate. Enrichment positive samples were assigned the direct plating detection limit of 250 CFU/g.

##### 2.6.1.2 Enumeration through quantitative polymerase chain reaction

###### 2.6.1.2.1 Deoxyribonucleic acid extraction and quantification from cecal contents

The *S.* Enteritidis load in the gut of the challenged birds was determined based on the detection of a highly specific gene (s*ef*-A) as previously described (33). Briefly, DNA was extracted from 0.2 to 0.3 g of cecal content of birds collected at DPC8 using QIAamp^®^ PowerFecal^®^ Pro DNA Kit (Qiagen GmbH, Hilden, Germany) following the manufacturer’s protocol. The DNA was eluted with 35 µL of the elution buffer for optimum DNA concentration. The concentration and purity of the extracted DNA were determined using a spectrophotometer (NanoDrop 2000c, Thermo Fisher Scientific, MA, USA). The A260/A280 nm absorbance ratio of ≤2.0 was considered indicative of pure DNA. The extracted DNA aliquots were stored at -20 °C until used in qPCR. Similarly, DNA was extracted from cultured *S.* Enteritidis and served as the positive control, while nuclease-free water was used as the negative control.

###### 2.6.1.2.2 Development of a calibration curve for *Salmonella* load estimation

A calibration curve was developed to estimate *Salmonella* load in the cecum of birds. Briefly, *S.* Enteritidis was cultured on XLT4 supplemented with nalidixic acid and novobiocin antibiotics. *S.* Enteritidis was inoculated on XLT4 media plates and incubated for 6 h at 37 °C. A distinct single colony with black pigment morphology typical to *Salmonella* culture was selected and inoculated in 50 mL TSB medium and incubated for 12 h at 37 °C. An aliquot (1 mL) of culture was re-inoculated into 100 mL of TSB broth and incubated at 37 °C under agitation (80RPM). The growth of the culture was monitored for 4 to 5 h until OD_600_ reached 0.65 and 0.70, measured in triplicates in a 96-well flat-bottom microtiter plate using a UV-spectrophotometer plate reader, with sterile TSB broth used as the blank. Similarly, to confirm presumptive *Salmonella* population in control samples, cultures of *S*. Enteritidis were serially diluted 10-fold and plated on XLT4 media plates supplemented with nalidixic acid and novobiocin. The plates were incubated for 24 h, and the culture colonies were enumerated to confirm the colony population. From the stock cultures of *S.* Enteritidis (Log 9 CFU/mL), DNA was extracted using QIAamp^®^ PowerFecal^®^ Pro DNA Kit (Qiagen GmbH, Hilden, Germany) from 10-fold dilutions of the stock cultures to a power of 1 (Log 1 CFU/mL). The qRT-PCR was performed as described below, and C_t_ values obtained from each culture dilution were plotted against the *Salmonella* colony population (known). A calibration curve developed in this study (*R*^2^ = 99%) was used to extrapolate the *S.* Enteritidis population in cecum expressed as logarithmic CFU per gram of ceca pools in birds.

###### 2.6.1.2.3 Real-time PCR to quantify *Salmonella* load

RT-PCR was performed to amplify *sef*A gene which codes for a major subunit protein of the fimbrial structure in *S.* Enteritidis (34, 35). The primer pair synthesized from integrated DNA Technologies was used to target s*ef*A gene in *S.* Enteritidis; *S*. Enteri-F (5′-GCA GCG GTT ACT ATT GCA GC-3′) and *S.* Enteri-R (5′-CTG TGA CAG GGA CAT TTA GCG-3′). The concentration of extracted DNA was adjusted to 10 ng using nuclease-free water. RT-PCR was performed in Applied Biosystems Quantistudio 5 Real-Time PCR system (Thermo Fisher Scientific, MA, USA) in a 20-µL total reaction volume. Each reaction cocktail was composed of 10 µL of 1× Quantabio PerfeCTa^®^ SYBR Green Fastmix (Quanta Biosciences Inc., Gaithersburg, MD, USA), forward and reverse primers (1 µL each), DNA template (2 µL of 10 ng), and nuclease-free water. The PCR condition was as follows: 95 °C for 15 s, 40 cycles of 95 °C for 15 s, and annealing for 30 s at 58 °C. The negative control cocktail was devoid of DNA template. *Salmonella* load in the cecal content of challenged birds was estimated by fitting the Ct values to a standard curve generated from serial dilutions of known concentrations of *S.* Enteritidis template.

#### 2.6.2 Qualitative estimation of *S.* Enteritidis in the reproductive tract

The upper oviduct (infundibulum and magnum) and lower oviduct (isthmus and uterus) from each chicken were collected during necropsy and kept on ice (36). An equal weight of PBS was added to each sample, and the mixture was homogenized using a tissue homogenizer. A 100-µL aliquot of each homogenized sample was plated directly onto XLT4 agar supplemented with nalidixic acid and novobiocin (20 µg/mL). The results were expressed as the absence or presence of *S.* Enteritidis based on the growth of black colonies on media plates.

#### 2.6.3 *Salmonella* testing in eggs

Eggs were collected twice daily (AM and PM) from *S.* Enteritidis-challenged birds. The PM-collected eggs were processed the following day along with the AM-collected eggs separately. All of the eggs were surface-disinfected with 70% ethanol and then aseptically broken. The albumen and yolk were carefully separated under sterile conditions. For each treatment group at a given time point, the yolk and albumen were pooled and homogenized in a Whirl-Pak bag. From each pooled sample, 1 mL of yolk and albumen was inoculated into TSB supplemented with 35 mg/L ferrous sulfate. Following the overnight incubation at 37°C, 20 µL of the enriched culture was transferred to Rappaport–Vassilidias (RV) broth and incubated at 42°C for 24 h. A loopful of this culture was streaked onto XLT4 agar supplemented with novobiocin and nalidixic acid (20 µg/mL), incubated for 24 h, and checked for the presence of black colonies (16, 37).

### 2.7 Antibody analysis by enzyme-linked immunosorbent assay

The OMP- and Fla-specific sIgA were measured in cloacal swab, bile, and small intestinal wash, and IgY antibodies were measured in serum and bile samples using enzyme-linked immunosorbent assay (ELISA) as described preciously (38). Briefly, the 96-well ELISA plates (Greiner Bio-one, Monroe, NC, USA) were coated overnight in coating buffer in duplicate wells with pre-titrated amounts of OMP and Fla proteins separately. For IgY estimation, OMP and Fla were used at 50 ng/well concentration, and for IgA estimation OMP and Fla were used at 50 and 400 ng/well, respectively. The plates were washed three times in PBS-T [1× PBS with 0.05% Tween-20 (Thermo Fisher Scientific, MA, USA)], and the non-specific binding sites were blocked using 5% non-fat dry milk diluted in PBS-T 200 µL/well for 2 h at room temperature (RT). The samples were diluted in 2.5% dry milk powder in PBS-T and incubated at 4 C overnight. The serum samples were diluted 1:200, whereas the bile samples were diluted 1:400 for detection of sIgA against OMP and flagella. For determining IgY levels in bile against OMP and Fla, dilutions of 1:400 and 1:200 were used, respectively. Cloacal swab and small intestinal wash were diluted 1:2. The specific secondary antibody, goat anti-chicken IgA peroxidase-labeled (Biorad, Kidkington, UK), or goat anti-chicken IgY (H+L) peroxidase-labeled (Southern Biotech, Birmingham, AL, USA) at pre-titrated dilution in 2.5% dry milk powder in PBS-T were added and incubated at RT for 2 h. The plates were washed thrice, and a 1:1 mixture of peroxidase substrate solution B and 3,3′,5,5′-tetramethylbenzidine (Thermo Fisher Scientific, MA, USA) was added. The reaction was stopped by adding 50 µL/well of 1 M phosphoric acid. The signal was read at OD_450_ nm using a microplate reader (SpectramaxPlus, Molecular Devices, Sunnyvale, CA, USA). The final OD was calculated by subtracting the average blank readings from the average readings of the duplicate test samples (25).

### 2.8 Extraction of antibodies in egg yolk and ELISA for the detection of OMP-specific IgY

Pooled yolk samples from eggs of each treatment group, collected daily post-challenge, were stored in 2 mL delipidation reagent at -20°C until further processing. Total IgY was extracted following the procedure described in Pierce Chicken IgY Purification kit (Thermo Scientific, MA, USA). Briefly, frozen samples were thawed, and 3 mL of additional delipidation reagent was added. The samples were mixed thoroughly and incubated overnight. Subsequently, they were centrifuged at 4,000 × *g* for 15 min, and the colorless supernatant was harvested. The process was repeated with a higher centrifugation speed for a longer duration until a clear supernatant was obtained. To the supernatant, an equal volume of cold IgY precipitation reagent was added and incubated overnight at 4°C. After centrifugation, the supernatant was discarded, and the resulting white pellet was dissolved in 1× PBS. The concentration of the antibodies in the samples was measured using a spectrophotometer (NanoDrop 2000c Spectrophotometer, ThermoFisher Scientific, MA, USA). To run the ELISA, OMP was coated with 100 ng/well in coating buffer. Three concentrations (20, 10, and 5 µg/50µl) of the isolated antibodies (IgY) were tested. Only the groups representing mock, mCS-NPs, and mCS(OMP+Fla)/Fla 200 µg dose were considered for the evaluation. The time points considered were pre-challenge (DPC0), DPC4 AM, and DPC8 AM. The remaining steps of the ELISA protocol were carried out as described above.

### 2.9 Quantitative real-time PCR for estimating immune cytokine gene expression

The RNA was extracted from the cecal tonsils by using the TRIzol method as described previously (38). Briefly, the quantity of RNA was measured using a spectrophotometer (NanoDrop 2000c Spectrophotometer, ThermoFisher Scientific, MA, USA). The extracted 2,000 ng RNA was used for cDNA synthesis. Reverse transcription template was made in a 20-μL reaction volume containing the reaction buffer. The procedure of cDNA synthesis involved denaturing the required RNA at 70°C for 12 min using a thermocycler (Mastercycler pro, Eppendorf, Germany). Each reaction of cDNA conversion contained 4 µL of M-MLV RT 5× buffer, 1 µL of 10 mM dNTPs, 0.5 µg of Oligo dT 15 primer, 100 units of M-LV reverse transcriptase, 8 units of recombinant RNAasin ribonuclease inhibitor (Promega, Madison, WI, USA), and 2 µL of 0.1 M dithiothreitol DTT (Invitrogen by Thermofisher Scientific, Carlsbad, CA, USA). Cytokine gene mRNA expression was quantified using Quantstudio 5 (Applied Biosystems by ThermoFisher Scientific, MA, USA). Each 20-µL reaction mixture comprised of 10 µL of PerfeCTa SYBR Green SuperMix (Quantabio, Beverly, MA, USA), 1 µL of cDNA (50 ng) template, and forward and reverse primers (2 µL each) with RNAse-free water added to make up the rest of the volume. The qPCR amplification protocol started with initial denaturation at 95 °C for 5 min (1 cycle), 40 cycles of 94 °C for 20 s, and gene-specific annealing temperature for 32 s. This was followed by these conditions sequentially: 95 °C for 15 s, 60 °C for 1 min, 95°C for 15 s, and 60°C for 15 s. The housekeeping gene β-actin was used to normalize the Ct values. Expression levels were normalized to the mock group and reported as fold change using the 2-^∆∆Ct^ method (39). The annealing temperatures, along with the sequences for the primers used, are provided in **Supplementary Table 1**.

### 2.10 Flow cytometry

Splenocytes were isolated from all of the birds on the day of necropsy using the Ficoll–Paque method, and the immunostaining protocol was followed as described previously (38). Briefly, 5 million cells per well were seeded in 1 mL complete Roswell Park Memorial Institute (RPMI) medium in a 48-well plate and stimulated with equal amounts of pooled OMP and Fla (10 μg/mL) incubated for 48 h. The cells (1 × 10^6^) were transferred to each of the 96-well round-bottom plates and washed (2,000 RPM for 2 min) once in 200 µL fluorescence-activated cell sorting (FACS) buffer/well. Fc receptor blocking was done by incubating with blocking buffer (1% normal mouse serum in FACS buffer) for 30 min at 4°C. After two PBS washes, the cells were stained for viability using the Fixable Dead Cell Stain Kit (Invitrogen, ThermoFisher Scientific) at 1:1,000 dilution in 1× PBS, with 50 µL/well for 30 min at 4°C. The cells were washed once in FACS buffer and proceeded for surface immunostaining using the fluorochrome labeled anti-chicken lymphocyte-specific antibodies or their corresponding isotype control antibodies at previously optimized concentrations in a final volume of 50 µL/well for 30 min at 4°C. Data were acquired on FACS Aria II (Becton, Dickinson and Company, NJ, USA) and analyzed using the FlowJo software (version 10). The details of the chicken antibody panels along with the gating strategy are provided in **Supplementary Table 2** and **Supplementary Figure 1**.

### 2.11 Statistical analysis

Statistical differences among groups were determined using one-way ANOVA, followed by Tukey’s multiple-comparison test using Prism 10 (GraphPad Software, Inc., CA, USA). Significant differences between treatment groups were established at *p* ≤ 0.05.

## 3 Results

### 3.1 Characterization of mannose-chitosan-based *Salmonella* subunit nanovaccine

To assess the quality of the extracted *Salmonella* antigens (OMP and Fla), sodium dodecyl sulfate–polyacrylamide gel electrophoresis (SDS-PAGE) was performed. For OMP, the major bands were observed at molecular weights of 37 and 28 kDa, whereas for Fla these were at approximately 52 kDa (40). Malvern Zetasizer was used to determine the size, PDI, and zeta potential of the nanoparticle vaccine formulation. The average size of the entrapped nanoparticle formulation was 252.1 ± 12.9, with polydispersity index of 0.4119 ± 0.088 and Zeta potential of 20.94 ± 0.1819 (**Table 2**).

**Table 2:** Physical nature of mChitosan–NP entrapped with OMP and Fla of *Salmonella* Enteritidis determined using Malvern Zetasizer.

Type of particles	Size (nm)	Polydispersity index	Zeta potential (+mV)
Empty particles	163.1 ± 1.163	0.26 ± 0.01	36.82 ± 0.535
mChitosan(OMP+Fla)	252.1 ± 12.9	0.4119 ± 0.088	20.94 ± 0.1819

### 3.2 Antibody responses against *Salmonella* antigen

#### 3.2.1 *Salmonella*-specific IgY responses

**Figure 2:**
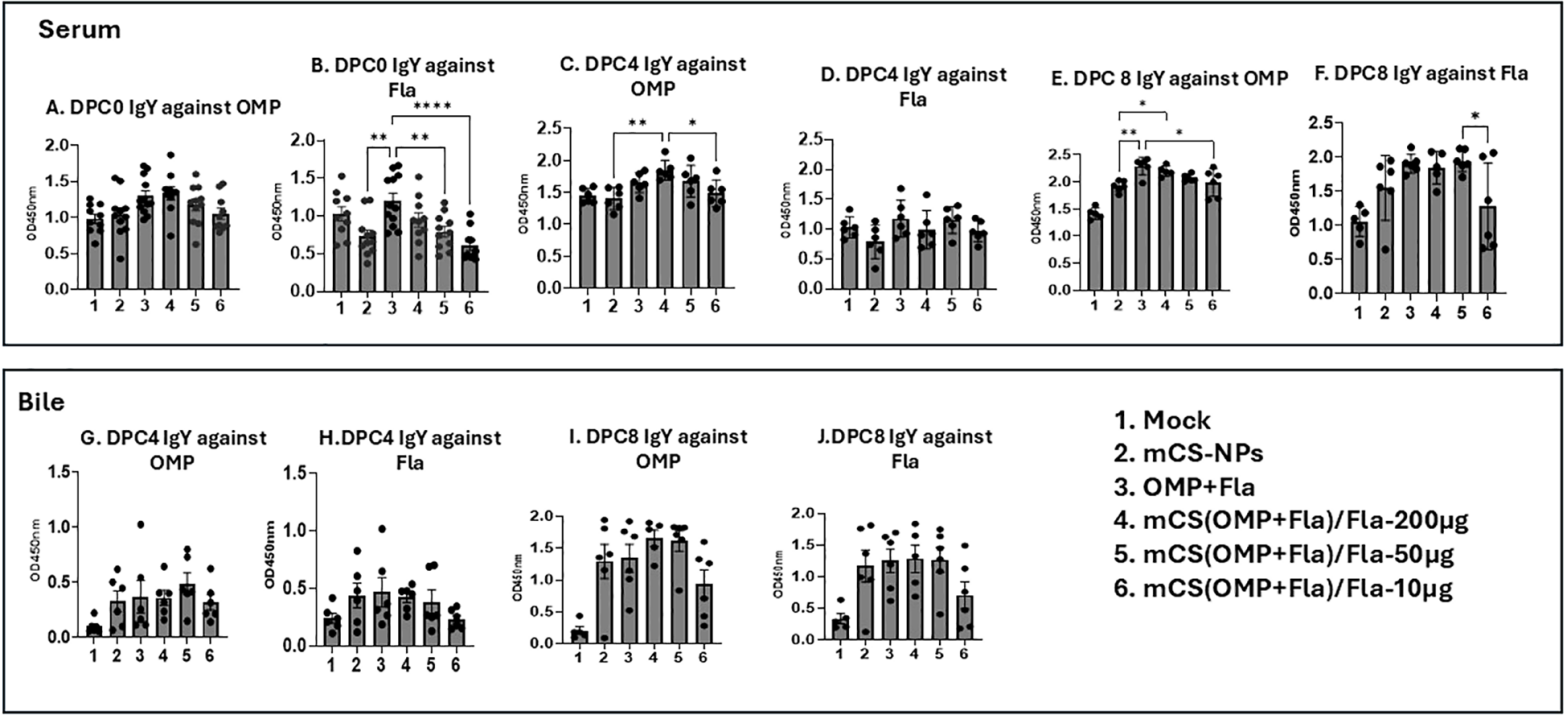
*Salmonella*-specific IgY antibody response in serum **(A–F)** and bile **(G–J)** at DPC0, DPC4, and DPC8. DPC, days post-challenge. Each bar is the mean ± SEM of five to 12 birds per group. The *P*-values between groups were determined by one-way ANOVA, followed by Tukey’s multiple comparisons *post-hoc* test. *p < 0.05, **p < 0.01, ****p < 0.0001.

Antigen-specific IgY response in the serum at pre-challenge (DPC0) showed a slight increase in mCS(OMP+Fla)/Fla-200 µg group compared to the other treatment groups (**Figure 2A**) against OMP. However, the OMP+Fla group showed significantly higher levels compared to mCS(OMP+Fla)/F-50 µg and mCS(OMP+Fla)/F-10 µg and the mCS-NPs group against Fla protein (**Figure 2B**). By 4 days post-challenge (DPC4), there was a statistically significant OMP-specific IgY level in the mCS(OMP+Fla)/Fla-200 µg group than the mCS-NPs and mCS(OMP+Fla)/Fla-10 µg groups (**Figure 2C**). At DPC8, the antibody titer levels had substantially increased, and the OMP+Fla vaccine group showed statistically significant OMP-specific IgY levels against OMP (**Figure 2E**). Against flagella OMP+Fla, mCS(OMP+Fla)/Fla-200 µg and mCS(OMP+Fla)/Fla-50 µg showed similar titers (**Figure 2F**). In the bile, OMP-specific IgY levels at DPC4 were relatively low compared to those in the serum (**Figures 2G, H**). However, by DPC8, the titers had increased, and mCS(OMP+Fla)/Fla-200 µg and mCS(OMP+Fla)/Fla-50 µg showed a slight increase in the response against OMP, but not against Fla (**Figures 2I, J**).

**Figure 3:**
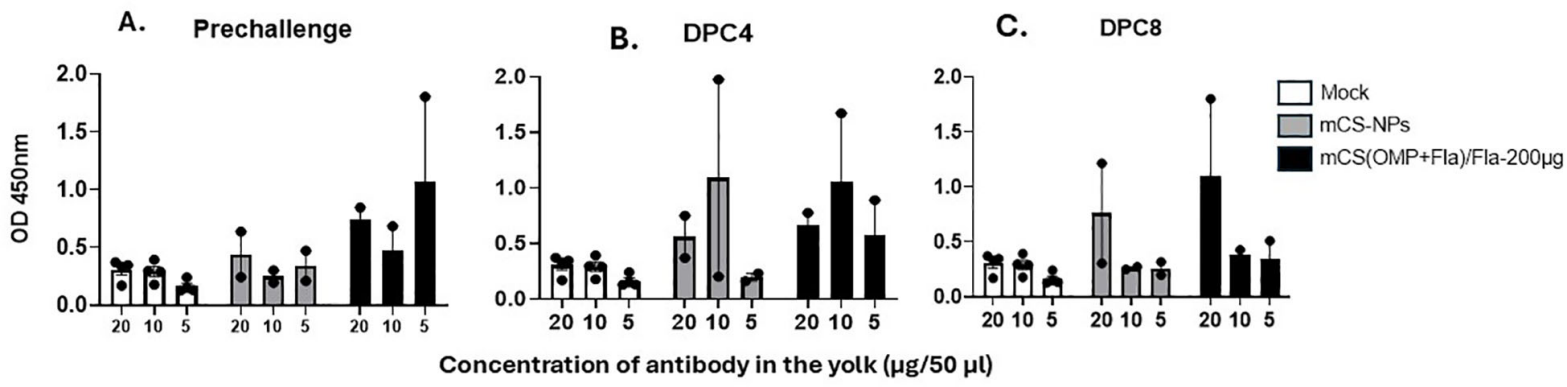
*Salmonella*-specific IgY response in the egg yolk collected on prechallenge (DPC0, **A**) DPC4 **(B)** DPC8 **(C)**. Total IgY from the egg yolk was extracted, and three indicated concentrations of total antibody were qualitatively tested against an optimized OMP concentration by ELISA. Each bar represents mean ± SEM indicative of antibody titer. For the mock group, birds necropsied at both time points DPC0 and DPC8 were combined.

OMP-specific IgY responses were also measured in the egg yolk. Our data indicated that the mCS(OMP+Fla)/Fla-200 µg vaccine group showed substantially higher IgY levels compared to the mock and mCS-NPs groups, particularly at DPC0 (**Figure 3A**). At DPC4, in the same group, a concentration of 5 µg/50 µL of isolated antibodies revealed a greater difference in IgY levels compared to the rest of the groups (**Figure 3B**). At DPC8, at a total antibody concentration of 20 µg/50µl, the mCS(OMP+Fla)/Fla-200 µg group showed numerically higher specific IgY responses compared to the control groups (**Figure 3C**).

#### 3.2.2 *Salmonella*-specific sIgA responses

**Figure 4:**
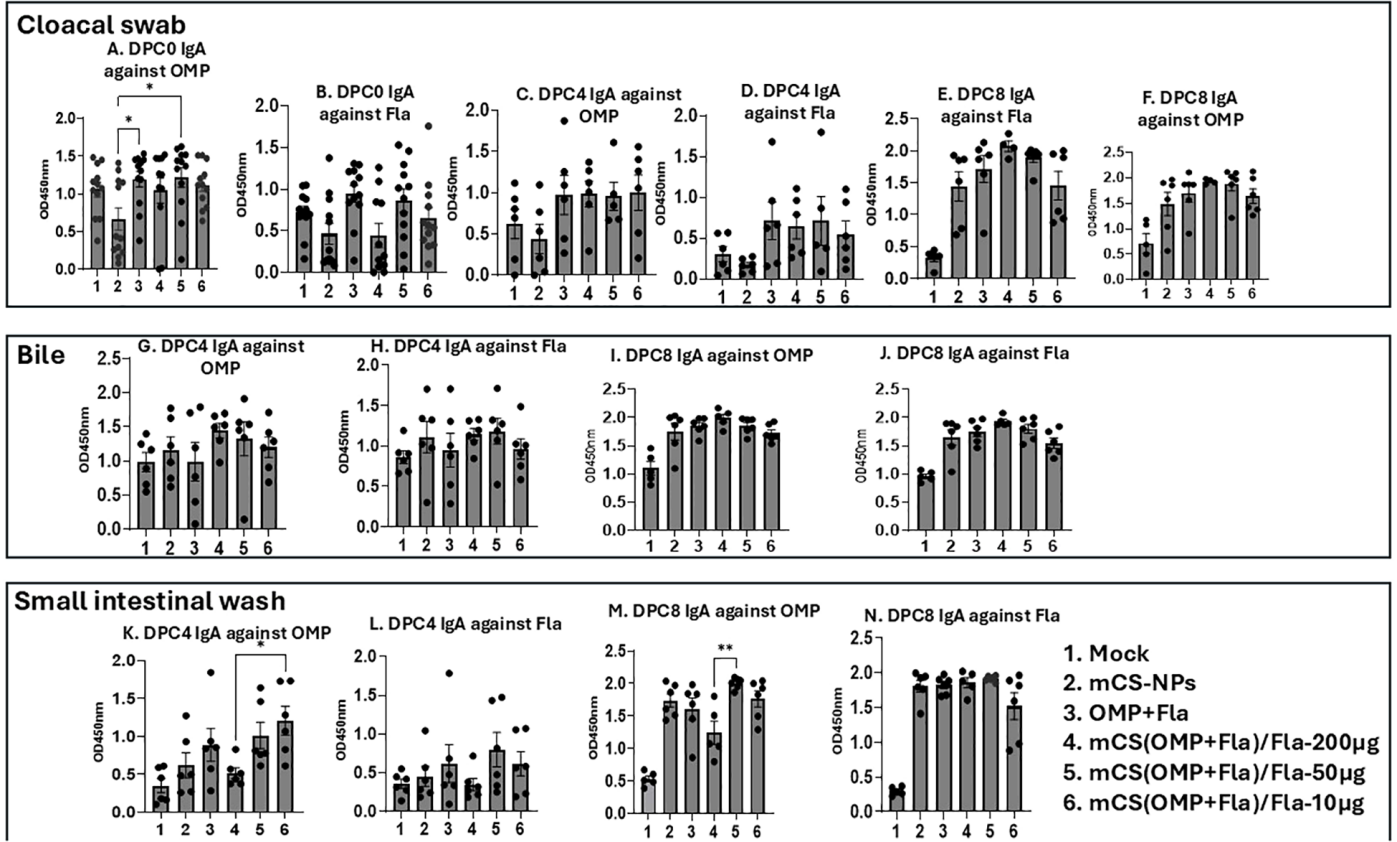
*Salmonella*-specific sIgA response in cloacal swab **(A–F)**, bile **(G–J)**, and small intestinal wash **(K–N)**. DPC, days post-challenge. Each bar is the mean ± SEM of five to 12 birds per group. The *P*-values between groups were determined by one-way ANOVA, followed by Tukey’s multiple comparisons *post-hoc* test. *p < 0.05, **p < 0.01.​.

In cloacal swabs at DPC0, the mCS(OMP+Fla)/Fla-50 µg and OMP+Fla vaccine groups showed significantly higher OMP-specific sIgA titers compared to the control mCS-NPs (**Figure 4A**). A similar numerical increase in sIgA against Fla was also observed, but the difference was not significant (**Figure 4B**). By DPC4, the specific sIgA titers against both OMP and Fla antigens were increased in all the nanovaccine including OMP+Fla groups (**Figures 4C, D**). However, by DPC8 antigen specific sIgA titers were relatively increased in mCS(OMP+Fla)/Fla-200 µg and mCS(OMP+Fla)/Fla-50 µg vaccine groups compared to rest of the groups (**Figures 4E, F**). In bile, a numerical increase in specific sIgA response was observed both at DPC4 and DPC8 in the mCS(OMP+Fla)/Fla-200 µg and mCS(OMP+Fla)/Fla-50 µg vaccine groups compared to the rest of the groups (**Figures 4G–J**). In the small intestine, however, the mCS(OMP+Fla)/Fla-10 µg vaccine group showed statistically higher sIgA levels against OMP on DPC4 (**Figure 4K**). The mCS(OMP+Fla)/Fla-50 µg group, compared to the -200 µg nanovaccine group, had statistically higher sIgA levels against OMP on DPC8 (**Figure 4M**).

### 3.3 Quantification of cytokine gene expression in the cecal tonsils

**Figure 5:**
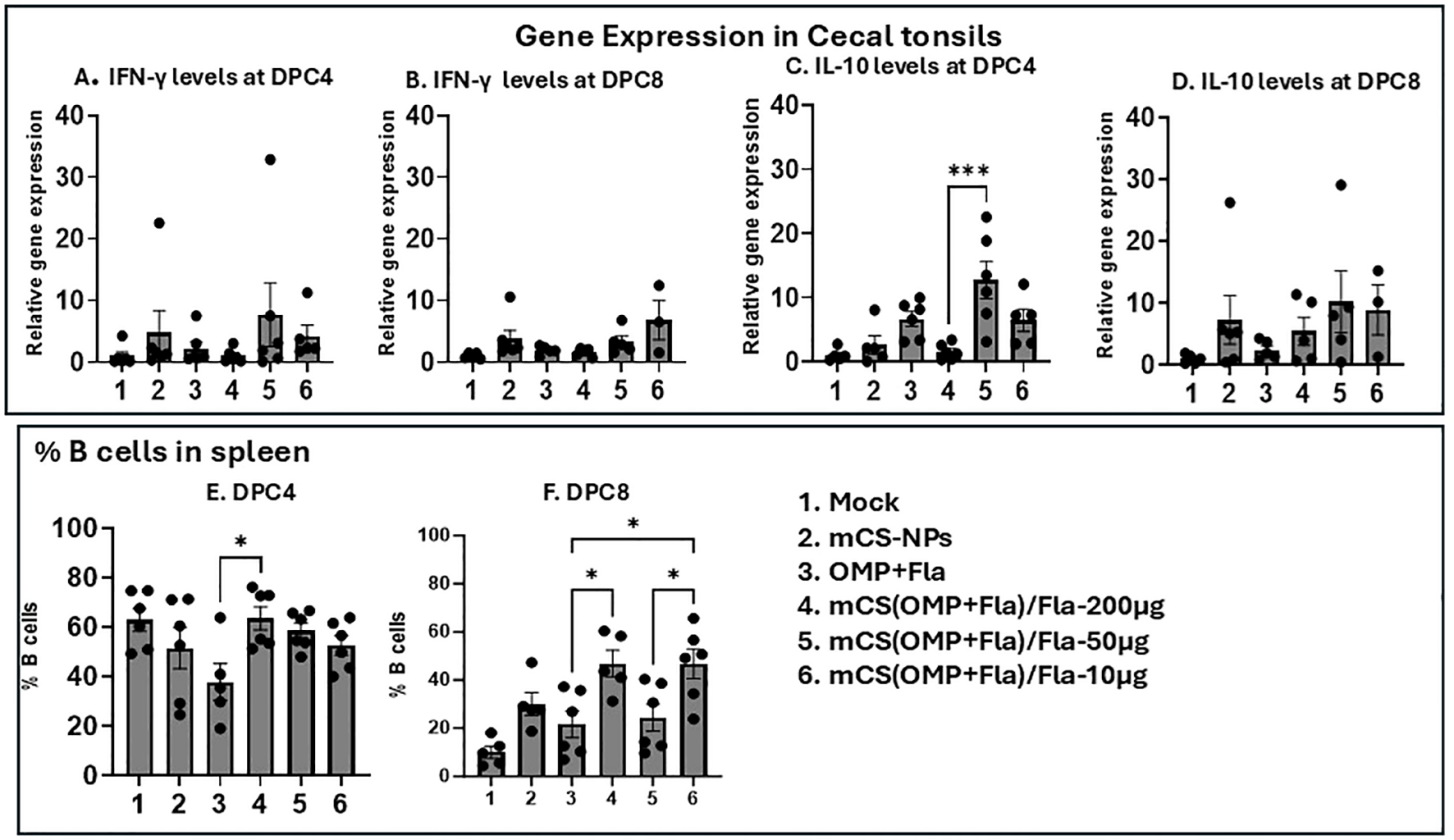
**(A–D)** Cytokine gene expression analysis in the cecal tonsils. IFN-γ **(A, B)**; IL-10 **(C, F)**. Each bar is the mean ± SEM of fold change in the gene expression of five to six birds per group. **(E, F)** Frequency of antigen-specific recall lymphocytes in the spleen. Cells were restimulated with pooled OMP+Fla for 48 h and subjected to flow cytometry analysis. Each bar is the mean ± SEM of percent B cells of five to six birds per group. The *P*-values between groups were determined by one-way ANOVA, followed by Tukey’s multiple comparisons *post-hoc* test. ​*p < 0.05, **p < 0.01, ***p < 0.001.

A relatively higher expression of IFN-γ gene was observed in the mCS(OMP+Fla)/Fla-50 µg and mCS(OMP+Fla)/Fla-10 µg nanovaccine groups on DPC4 and DPC8, respectively (**Figures 5A, B**). An increase in the IL-10 mRNA levels was statistically higher in the mCS(OMP+Fla)/Fla-50 µg group compared to the mCS(OMP+Fla)/Fla-200 µg nanovaccine group on DPC4 (**Figure 5C**), and a similar numerical increase was observed on DPC8, although not statistically significant (**Figure 5D**). The other cytokine genes analyzed include IL-17A, IL-4, and TGF-β, with no apparent increase among the groups (data not shown).

### 3.4 Modulation of antigen-specific B and T cell frequencies in nanovaccine-receiving birds

Flow cytometry analysis was performed to assess the cell-mediated immune response. Immune cell subsets such as TCRγδ T and B cells were analyzed in isolated splenocytes in a OMP+Fla antigen recall response. The frequency of B cells (CD3^-^Bu1^+^) was statistically higher in mCS(OMP+Fla)/Fla-200 µg on DPC4 and DPC8 compared to the OMP+Fla group (**Figures 5E, F**). Additionally, the mCS(OMP+Fla)/Fla-10 µg group also showed a statistically higher frequency of B cells on DPC8 compared to the OMP+Fla group (**Figure 5F**). However, we did not observe any vaccine-mediated response in the TCRγδ T cell subset (data not shown).

### 3.5 Challenge bacterial load in the ceca and reproductive tract of birds post-challenge

**Figure 6:**
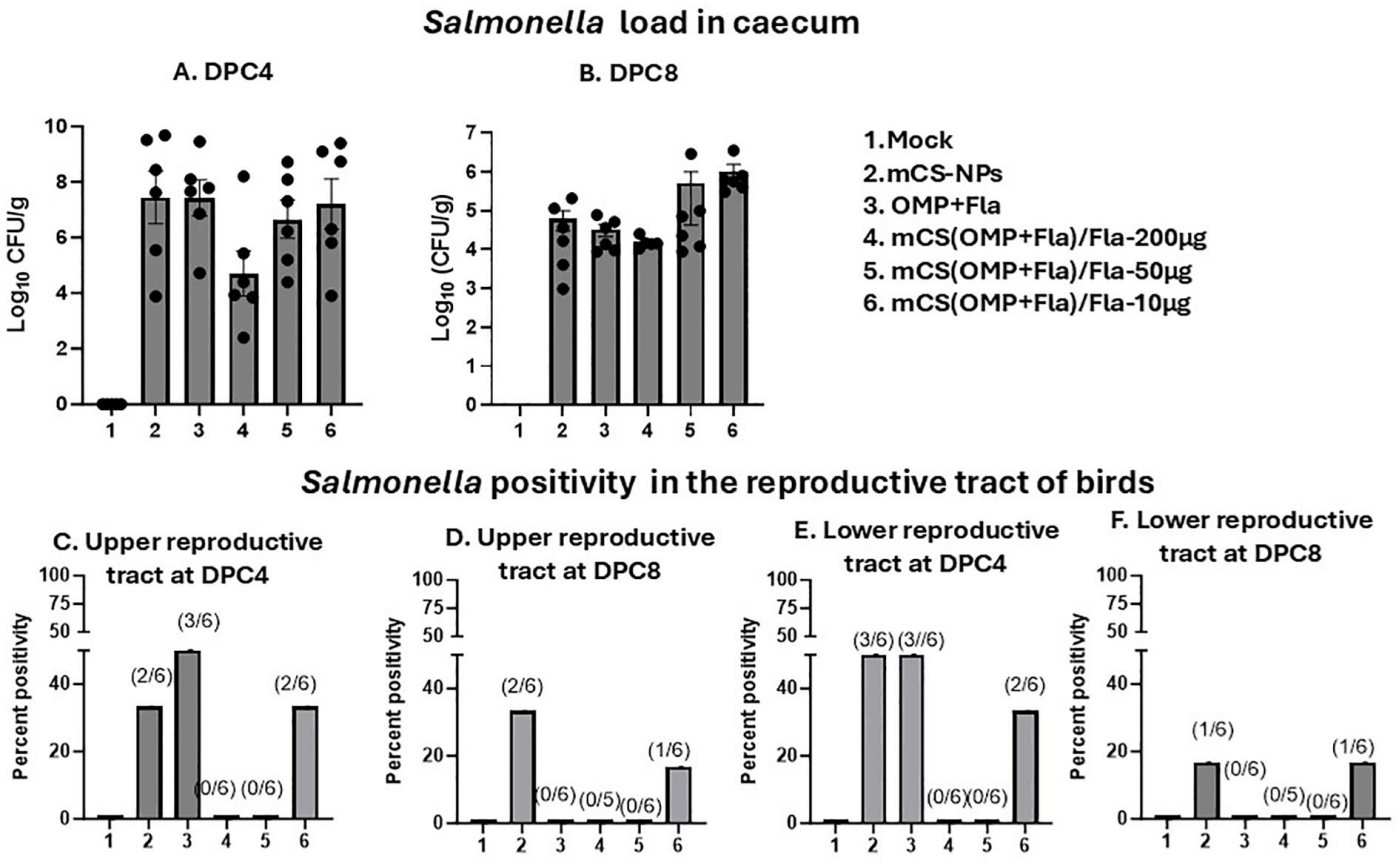
**(A)** Live *Salmonella* load in the cecum determined by standard culture plating method. **(B)** *Salmonella* genomic load determined by qPCR. Each bar represents the mean ± SEM of five to six birds per group. One-way ANOVA followed by Tukey’s multiple comparisons *post-hoc* test was conducted to determine the *P*-values, but there was no statistical difference observed between the groups. **(C–F)** *Salmonella* positivity in upper and lower reproductive tract at DPC4 and DPC8. DPC, days post-challenge. *Salmonella* positivity was descriptively compared between the groups due to less sample size and low expected frequencies.

The challenge *S.* Enteritidis load present in the ceca of birds was evaluated at both DPC4 and DPC8. Although the results were not statistically significant among the three nanovaccine dose groups, a substantial reduction in *Salmonella* load was observed in mCS(OMP+Fla)/Fla-200 µg with 2.74 log reduction compared to the control mCS-NPs group on DPC4. Similarly, in the mCS(OMP+Fla)/Fla-50 µg group, 0.79 log reduction was observed compared to the control mCS-NP group on DPC4. Interestingly, the control group receiving 200 µg of OMP+Fla soluble antigens not entrapped in NPs failed to reduce the *Salmonella* load in birds. Overall, a dose-dependent nanovaccine efficacy in reducing the challenge *Salmonella* load on DPC4 was observed in birds analyzed by the standard CFU plate count method (**Figure 6A**).

At DPC8, the standard plate count method did not reveal an appreciable log difference in the challenge *Salmonella* load among the vaccinated groups. Therefore, qPCR assay was employed to determine the *Salmonella* load at DPC8-collected samples. We observed a relatively reduced *Salmonella* load in the mCS(OMP+Fla)/Fla-200 µg vaccine group compared to the control groups, consistent with the findings observed at DPC4 (**Figure 6B**). However, numerically higher bacterial loads were detected by qPCR in birds vaccinated with the two lower doses, but the differences were not statistically significant. This observation may indicate the presence of potentially high numbers of dead bacterial DNA.

In the reproductive tract of mCS(OMP+Fla)/Fla-200 µg and mCS(OMP+Fla)/Fla-50 µg vaccine groups, the challenge *Salmonella* was completely cleared in all of the birds at both DPC4 and DPC8, both in the lower and upper reproductive tracts, using the standard direct plating method (**Figures 6C–F**). In contrast, in mCS(OMP+Fla)/Fla-10 µg vaccinates, one to two birds still had detectable *Salmonella* in the reproductive tract on both necropsy days (**Figures 6C–F**). In the OMP+Fla control vaccine group, *Salmonella* was detected in three out of six birds at DPC4 in the upper and lower reproductive tracts (**Figures 6C, E**), while by DPC8 complete clearance was noticed in both reproductive tracts (**Figures 6D, F**). These data were descriptively compared between the groups due to less sample size and low expected frequencies. Although the reproductive tract of control group birds had positivity for *Salmonella*, the bacteria was not detectable in the eggs tested everyday post-challenge infection.

## 4 Discussion

In this study, our goal was to evaluate the efficacy of mannose-conjugated chitosan nanoparticles encapsulating *Salmonella* antigen vaccine in adult layer chickens raised under a specific-pathogen-free (*Salmonella*) condition. This work builds upon our previous study in layers terminated at 16 weeks of age, where the same *Salmonella* nanovaccine was similarly administered 3-times orally (21, 24). In layer chickens, it is important to evaluate the efficacy of *Salmonella* vaccines at the onset of egg laying, as the incidence of salmonellosis is higher during that period. This is because, as the pullets reach sexual maturity, there is a drop in the frequency of T-helper cells and γδ-T lymphocytes, thus causing a reduction in immune protection. This immunological shift is responsible for the increased susceptibility of pullets to *Salmonella* (41).

Additionally, unlike the typical termination of broilers at 6 weeks of age, layers are kept for production for up to 2 to 3 years. Thus, it is important to evaluate the efficacy of a vaccine for long-lasting immunity. The routinely used orally administered live *S.* Typhimurium vaccine failed to induce long-lasting protection in layers (42). Another important consideration for a vaccine intended for farm animals, especially for poultry, is their scalability and ease of administration to large poultry flocks in commercial settings. Our candidate *Salmonella* nanovaccine has been developed for oral delivery, particularly for administration through drinking water, which is user friendly and less labor-intensive. Chitosan is very inexpensive, and thus the formulation is cost-effective. Furthermore, the vaccine is in freeze-dried form and stable for several months to years when stored at 4 °C.

Chitosan was used to prepare our nanovaccine because of its numerous advantages as a mucosal vaccine delivery platform. It is a biodegradable and biocompatible polysaccharide polymer with strong mucoadhesive properties. It particularly interacts with the nasal and intestinal epithelium due to its high positive charge, prolonging the contact time of nanovaccine particles with the epithelium, enhancing their uptake particularly by dendritic cells, and promoting their activation (43). Chitosan induces the transient opening of epithelial tight junctions between Caco-2 cells, facilitating the paracellular transport of vaccine cargo when delivered in nanoparticles (44). The other agent included in our nanovaccine formulation is mannose, which interacts with the mannose receptor on dendritic cells and macrophages, facilitating the targeted endocytosis and phagocytosis of vaccine particles (45, 46). The molecular size of OMP and Fla proteins of *S.* Enteritidis used in our nanovaccine formulation closely matched that of an earlier report (40). The physical properties of the nanovaccine revealed that the charge was 20.94 ± 0.1819 mV, the average size of NPs was 252.1 ± 12.9 nm, and the PDI was 0.4119 ± 0.088, indicating that the nanovaccine formulation has ideal physical properties for uptake by dendritic cells and macrophages via active endocytosis (47). The PDI measures the uniformity of the nanovaccine particles, and a value <0.5 is acceptable for a vaccine, which signifies a more uniform size distribution (48).

Antibody responses were detected in *Salmonella*-nanovaccine-administered birds at different time points, and a gradual increase in specific IgY levels in serum from DPC0 to DPC8 was observed. The OMP-specific IgY was also increased in the egg yolk of mCS(OMP+Fla)/Fla-200 µg vaccinates. The sIgA response is one of the most important markers for mucosal immunity (49). In our vaccinates, particularly the mCS(OMP+Fla)/Fla-200 µg and mCS(OMP+Fla)/Fla-50 µg dose groups, a substantial production of specific sIgA in the intestines and bile was observed. Consistent with the antibody production, an increased population of antigen-specific B cells in a recall response of splenocytes was observed; the memory B cells contribute to enhanced recall responses in chicken (50, 51). Activated B cells induce antibody class switching and help in memory development by recognizing *Salmonella* antigens via B cell receptors, present the antigenic peptides via MHC class II molecules, and interact with follicular T-helper cells (52).

*S.* Enteritidis does not cause any sign of disease in birds. Hence, it is hard to demonstrate the protective efficacy of a vaccine. Thus, the evaluation of a vaccine’s efficacy relies mainly on the reduction of bacterial colonization in the intestines of birds (52). Additionally, most experimental studies in adult layers use an oral challenge dose of 10^9^ CFU, and even that high dose results in very low levels of the bacteria in the eggs of healthy adult birds (53). It is noteworthy to mention that the contamination level of eggs with *S.* Enteritidis is proportional to the oral dose (53–55). This necessitates a high-challenge dose, like 10^9^ CFU per bird, which also causes reproductive tract infection (53, 55, 56). Furthermore, challenge of birds at the age of sexual maturity has also been shown to result in higher colonization rates of *S.* Enteritidis in the intestines and reproductive tract of layers (57). However, a high challenge dose in birds could compromise vaccine efficacy by potentially eliciting enhanced antibody responses early after challenge infection (58).

Furthermore, we observed a consistent increase in gene expression of cytokines involved in immune modulation, such as IFN-γ and IL-10, in the cecal tonsils of mCS(OMP+Fla)/Fla vaccinates that received two lower doses. IFN-γ is involved in T-helper 1-associated responses, promoting antigen presentation and processing of intracellular antigens (59). Cytokine IL-10 is involved in immune homeostasis and has been suggested as a potential target for improving vaccine efficacy (60). Despite detecting a robust reduction in the challenge bacterial load at DPC4, the absence of a stronger cytokine response in the mCS(OMP+Fla)/Fla-200 µg vaccine group suggested the need to assess the cytokine levels at early time points post-infection.

One of the primary goals of this study was to determine if *Salmonella* contamination of eggs could be reduced through vaccination. Experimental studies have shown much lower occurrences of *Salmonella* in eggs, typically <7%, with the yolk being frequently contaminated than the albumen (55, 61–63). Furthermore, factors within eggs could influence the growth of pathogens before or after laying (64). Additionally, egg samples from each group were pooled, as low positivity rates were expected. However, we do acknowledge that this approach may have resulted in reduced sensitivity. We adapted this method due to our practical difficulties in handling and processing many egg samples daily. In our future studies, we plan to incorporate additional diagnostic modalities such as PCR, alongside conventional culture methods, including two-step enrichment of egg samples in two types of broth before plating to improve the sensitivity of bacterial detection. Since none of the eggs in our trial, even in unvaccinated challenge birds, was found positive for *Salmonella*, we performed a detailed analysis of both the upper (infundibulum and magnum) and lower (isthmus and uterus) reproductive tracts separately by culturing the homogenized tissues for qualitative estimation of *Salmonella* because the bacteria can be transmitted vertically from the reproductive tract to the eggs (11, 53, 55). In birds vaccinated with mCS(OMP+Fla)/Fla-200 µg and mCS(OMP+Fla)/Fla-50 µg, *Salmonella* was not detected in the reproductive tract by standard plating method at both necropsy days (DPC4 and DPC8).

In the ceca, 2.75 log reduction in the challenge *S.* Enteritidis load in mCS(OMP+Fla)/Fla-200 µg vaccinates at DPC4 supports that the orally delivered nanovaccine substantially reduced the bacterial load in the intestines; furthermore, at DPC8, the qRT-PCR data were in support of this observation. These data should be approached with caution as qPCR detects both viable and non-viable bacterial DNA (65). Therefore, the increased *Salmonella* load in the lower-dose groups might not be reflective of the true bacterial load. In our future studies, we will consider using ethidium monoazide PCR to differentiate viable and dead bacteria as described previously (66). Interestingly, we could achieve a much higher reduction (1 log vs 2.75 log) in the *Salmonella* clearance than in our earlier studies in layers (21). This has been attributed to the additional booster vaccination performed in this trial and the difference in the age of the birds that received the vaccine (5, 8, and 11 weeks of age versus 4, 10, 16, and 18 weeks of age). Reduced cecal *Salmonella* loads have been associated with decreased carcass contamination in both pigs and poultry (67, 68). In addition, a study indicated that *Salmonella* present in the gut can translocate and persist in internal organs, contributing to foodborne risk (69). Therefore, the decreasing *Salmonella* levels in the intestinal content likely reduced the bacterial burden in other organs, including in the reproductive tract, and, in turn, the risk of transmission. Mathematically, 1- log reduction in *Salmonella* load corresponds to a 90% decrease in bacterial load, while a 2- log reduction means 99% decrease (70). Thus, in this study, the nanovaccine was able to provide a biologically meaningful mitigation of *Salmonella* burden in layer birds. This was indicated by a nearly 3-log reduction in the intestinal load and absence of detectable *Salmonella* by direct plating in the reproductive tract of the nanovaccine-administered adult layers in a dose-dependent manner.

## 5 Conclusion

**Figure 7:**
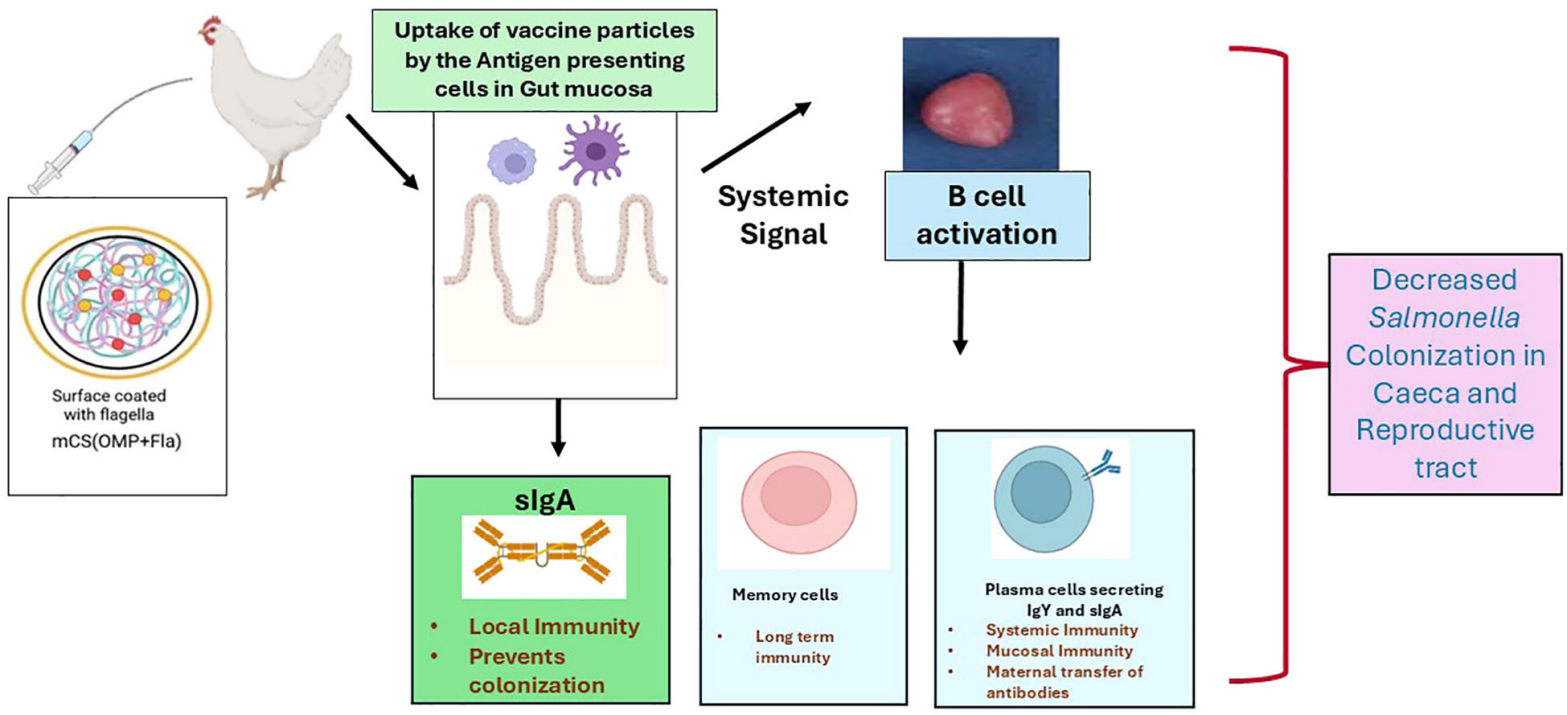
Conclusion of the study. Created with Biorender.com.

This study evaluated for the first time the efficacy of three different doses of mannose–chitosan nanoparticle-based *Salmonella* subunit vaccine in adult layer chickens, administered four times orally, and determined the immune correlates of protection. Our data revealed that *Salmonella* load was substantially reduced in mCS(OMP+Fla)/Fla vaccinates in a dose-dependent manner, which was observed both in the intestines and the reproductive tract. Immunologically, the efficacy was associated with elevated specific sIgA and IgY responses at both systemic and mucosal sites, including in the egg yolk, and enhanced specific memory B cell responses (**Figure 7**). These findings suggest that the chitosan nanoparticle-based *S.* Enteritidis subunit vaccine has a potential to lower the risk of *Salmonella* in eggs.

## Data Availability

The original contributions presented in the study are included in the article/**Supplementary Material**. Further inquiries can be directed to the corresponding author.

## Glossary

CDC: Centers for Disease Control and Prevention
FDA: Food and Drug Administration
OMP: outer membrane proteins
Fla: flagella
TSB: Tryptic soy broth
OD: optical density
MicroBCA: micro bicinchoninic acid assay
BHI: brain heart infusion (broth)
mCS-NPs: mannose-modified chitosan nanoparticles
RPM: revolutions per minute
PBS: phosphate-buffered saline
MOPS: 3-(-N-morpholino) propanesufonic acid
TPP: sodium tripolyphosphate
PDI: polydispersity index
SPF: specific pathogen free
CFU: colony-forming unit
DPC: days post-challenge
IgY: immunoglobulin Y
sIgA: secretory immunoglobulin A
XLT4: xylose lysine tergitol-4
DNA: deoxyribonucleic acid
qPCR: quantitative polymerase chain reaction
Ct value: cycle threshold value
RV Broth: Rappaport–Vassiliadis broth
ELISA: enzyme-linked immunosorbent assay
PBS-T: phosphate-buffered saline with Tween 20
RNA: ribonucleic acid
cDNA: complementary deoxyribonucleic acid
mRNA: messenger ribonucleic acid
RPMI: Rosewell Park Memorial Institute
FACS: fluorescence-activated cell sorting
SDS-PAGE: sodium dodecyl–polyacrylamide gel electrophoresis.
